# The K^+^ Channel K_Ca_3.1 as a Novel Target for Idiopathic Pulmonary Fibrosis 

**DOI:** 10.1371/journal.pone.0085244

**Published:** 2013-12-31

**Authors:** Katy Morgan Roach, Stephen Mark Duffy, William Coward, Carol Feghali-Bostwick, Heike Wulff, Peter Bradding

**Affiliations:** 1 Institute for Lung Health, Department of Infection, Immunity and Inflammation, University of Leicester, Leicester, United Kingdom; 2 Division of Respiratory Medicine, Centre for Respiratory Research and Nottingham Respiratory Biomedical Research Unit, University of Nottingham, Nottingham, United Kingdom; 3 Department of Medicine, Division of Pulmonary, Allergy, and Critical Care Medicine, University of Pittsburgh, Pittsburgh, Pennsylvania, United States of America; 4 Department of Pharmacology, University of California Davis, Davis, California, United States of America; Sackler Medical School, Tel Aviv University, Israel

## Abstract

**Background:**

Idiopathic pulmonary fibrosis (IPF) is a common, progressive and invariably lethal interstitial lung disease with no effective therapy. We hypothesised that K_Ca_3.1 K^+^ channel-dependent cell processes contribute to IPF pathophysiology.

**Methods:**

K_Ca_3.1 expression in primary human lung myofibroblasts was examined using RT-PCR, western blot, immunofluorescence and patch-clamp electrophysiology. The role of K_Ca_3.1 channels in myofibroblast proliferation, wound healing, collagen secretion and contraction was examined using two specific and distinct K_Ca_3.1 blockers (TRAM-34 and ICA-17043 [Senicapoc]).

**Results:**

Both healthy non fibrotic control and IPF-derived human lung myofibroblasts expressed K_Ca_3.1 channel mRNA and protein. K_Ca_3.1 ion currents were elicited more frequently and were larger in IPF-derived myofibroblasts compared to controls. K_Ca_3.1 currents were increased in myofibroblasts by TGFβ1 and basic FGF. K_Ca_3.1 was expressed strongly in IPF tissue. K_Ca_3.1 pharmacological blockade attenuated human myofibroblast proliferation, wound healing, collagen secretion and contractility *in* *vitro*, and this was associated with inhibition of TGFβ1-dependent increases in intracellular free Ca^2+^.

**Conclusions:**

K_Ca_3.1 activity promotes pro-fibrotic human lung myofibroblast function. Blocking K_Ca_3.1 may offer a novel approach to treating IPF with the potential for rapid translation to the clinic.

## Introduction

Idiopathic pulmonary fibrosis (IPF) is a progressive fibrosing interstitial pneumonia of unknown etiology [1]. The term IPF is now restricted to patients with radiographic features consistent with the histological pattern of usual interstitial pneumonia (UIP). It occurs primarily in older adults [1] with an incidence of 16 per 100,000 person-years in the USA [2]. In the UK there are over 4,000 cases diagnosed annually, which is an equivalent disease burden with that of ovarian and kidney cancers [3]. There is no effective treatment[1] and prognosis is poor with a median survival of only 2-3 years from diagnosis [3]. IPF therefore represents an important cause of morbidity and mortality and novel approaches to treatment are required urgently to address this unmet clinical need. 

The pathogenic mechanisms involved in IPF initiation and progression are poorly understood [4]. Myofibroblasts play a critical role in tissue repair through cell-cell and cell-matrix interactions [5], maintaining and regulating extracellular matrix, interstitial fluid volume, and the extent of tissue contraction needed for optimum function [6]. However, dysregulated or inappropriate myofibroblast function leads to pathological scarring and tissue fibrosis [7]. The myofibroblast is the principle cell responsible for the synthesis and deposition of the fibrotic matrix in IPF and the associated tissue contraction [6]. Targeting pro-fibrotic myofibroblast activity therefore offers the potential to slow down or halt the progression of IPF. 

Ion channels are attractive therapeutic targets in many chronic diseases. The Ca^2+^ activated K^+^ channel K_Ca_3.1 plays an important role in Ca^2+^ signalling through its ability to maintain a negative membrane potential during cell activation [8]. The K_Ca_3.1 channel modulates the activity of several structural and inflammatory cells, including lymphocytes [9], mast cells [10], and dedifferentiated smooth muscle cells [11], through the regulation of cell proliferation [11], activation [9], migration [10] and mediator release [12]. Pharmacological inhibition or genetic deletion of K_Ca_3.1 prevents surgically induced renal fibrosis in mice by targeting myofibroblasts, leading to reduced collagen deposition and fibroblast proliferation while preserving renal parenchyma [13]. 

Both TGFβ1 and basicFGF are key growth factors which drive myofibroblast-dependent fibrosis in IPF [5,6]. We hypothesise that TGFβ1- and basicFGF-driven K_Ca_3.1-dependent cell processes are a common denominator in the pathophysiology of IPF. In this study we have investigated the expression and function of the K_Ca_3.1 channel in primary human lung myofibroblasts derived from both non-fibrotic and IPF lungs.

## Materials and Methods

### Ethics statement

All patients donating tissue gave written informed consent and the study was approved by the National Research Ethics Service (references 07/MRE08/42 and 10/H0402/12).

### Human lung myofibroblasts isolation and culture

Non-fibrotic control (NFC) myofibroblasts were derived from healthy areas of lung from patients undergoing lung resection for carcinoma at Glenfield Hospital. No morphological evidence of disease was found in the tissue samples used for myofibroblast isolation. IPF myofibroblasts were derived from patients undergoing lung biopsy for diagnostic purposes at the University of Pittsburgh Medical Center, and were shown to have UIP on histological examination. Myofibroblasts were grown from explanted lung tissue from both sources under identical conditions, using Dulbecco’s modified Eagle’s medium (DMEM) supplemented with 10% fetal bovine serum (FBS), antibiotic/antimycotic agents and non-essential amino acids [14,15]. The cells were cultured at 37°C in 5% CO_2_/95% air. Cells were studied at passages 4-5 for functional studies. All NFC patients gave informed written consent and the study was approved by the Leicestershire, Northamptonshire and Rutland Research Ethics Committee 2. Written informed consent was also obtained from all IPF subjects, in accordance with the responsible University of Pittsburgh Institutional Review Board.

### Human myofibroblast characterisation using immunofluorescent staining

Human myofibroblasts were harvested from 80-90% confluent monolayers with 0.1% trypsin/0.1% EDTA. Cells were seeded into 8-well chamber slides, grown to confluence, and immunostained using the following antibodies: FITC-conjugated mouse monoclonal anti-α-smooth muscle actin (αSMA) (F3777, 10 µg/ml, Sigma-Aldrich, Poole, Dorset, UK) and isotype control FITC-conjugated mouse IgG_2a_ (X0933, 10 µg/ml, Dako, Ely, UK); mouse monoclonal anti-fibroblast surface protein(FSP)(F4771, 4 µg/ml, Sigma-Aldrich) and isotype control mouse IgM (M5909, 4 µg/ml, Sigma-Aldrich); mouse monoclonal anti-fibroblast antigen THY-1 (CP28, 3 µg/ml, Calbiochem, San Diego, CA) and isotope control IgG_1_ (X0931, 3µg/ml, Dako); rabbit polyclonal anti-K_Ca_3.1 (P4997, 8 µg/ml, Sigma-Aldrich; M20, a gift from Dr M Chen, GlaxoSmithKline, Stevenage, UK), isotype control rabbit IgG (550875, BD Pharmagen, 8 µg/ml). Rabbit polyclonal collagen type 1 (550346, 20 µg/ml, Millipore, Watford, UK), isotype control rabbit IgG (20 µg/ml). Monoclonal mouse CD68 antibody (6.4 µg/ml, Dako), isotype control IgG1 and CD34 R-PE antibody (0.5 µg/ml, Catlag) and isotype control IgG1 R-PE was also used. Secondary antibodies labelled with FITC or R-PE (F0313, Dako) were applied and the cells counterstained with 4',6-diamidino-2-phenylindole (DAPI, Sigma-Aldrich). Cells were mounted with fluorescent mounting medium and cover-slipped. Original images were captured on an epifluorescent microscope (Olympus BX50, Olympus UK Ltd, Southend–on-sea) and counted using Cell F imaging software (Olympus UK Ltd). Matched exposures were used for isotype controls.

### Immunohistochemistry on human lung tissue

Sections were cut at 4 µm from paraffin-embedded diagnostic lung parenchymal biopsies or healthy control lung obtained at cancer surgery, and immunostained using mouse anti-αSMA mAb (1A4, 0.7 µg/ml, Dako), rabbit polyclonal anti-K_Ca_3.1 (AV35098, 5 µg/ml, Sigma-Aldrich), or appropriate isotype control at the same concentration as the primary antibody. 

### qRT-PCR

Myofibroblast RNA was isolated using the RNeasy Plus Kit (Qiagen, West Sussex, UK) according to the manufacturer’s instructions. K_Ca_3.1 gene expression was analyzed with the gene-specific Quantitect Primer Assay (Qiagen, Hilden, Germany), Hs_KCNN4_1_SG. The internal normaliser gene used was β-actin (forward primer, 5’-TTCAACTCCATCATGAAGTGTGACGTG-3’, reverse primer, 5’- CTAAGTCATAGTCCGCCTAGAAGCATT-3’). All expression data was normalized to β-actin and corrected using the reference dye ROX. Gene expression was quantified by real-time PCR using the Brilliant SYBR Green QRT-PCR 1-Step Master Mix (Strategene, Amsterdam, The Netherlands). PCR products were run on a 1.5% agarose gel to confirm the product amplified was the correct size: K_Ca_3.1 130 base pairs (bp), β-Actin 310 bp.

To study the effects of either TGF-β1 (10 ng/ml, R&D Systems, Abingdon, UK) or bFGF (10 ng/ml, R&D Systems) on K_Ca_3.1 mRNA expression, cells were grown to confluence and then serum starved for 24 h prior to growth factor exposure for 24 h.

### Western blot

Human myofibroblasts were disrupted in lysis buffer and soluble proteins from equivalent numbers of cells were resolved by 12% SDS-PAGE and then transferred to an immunobilon- P polyvinylidene difluoride membrane. Membranes were blocked and incubated with rabbit polyclonal K_Ca_3.1 antibodies (Sigma antibodies P4997, AV35098 and GSK gift antibody M20. Protein bands were identified by horseradish peroxidase-conjugated secondary antibody and enhanced chemiluminescence reagent (Amersham, Little Chalfont, UK). Immunolabelled proteins were visualized using an ECL western blot detection system (GE Healthcare Life Sciences, Buckinghamshire, UK).

### Patch clamp electrophysiology

The whole cell variant of the patch clamp technique was used as described previously[16,17]. The standard pipette solution contained (in mM) KCl, 140; MgCl_2_, 2; HEPES, 10; Na^+^-ATP, 2; GTP, 0.1 (pH 7.3). The standard external solution contained (in mM) NaCl, 140; KCl, 5; CaCl_2_, 2; MgCl_2_,1; HEPES, 10 (pH 7.3). For recording, myofibroblasts were placed in 35-mm dishes containing standard external solution. Whole-cell currents were recorded using an Axoclamp 200A amplifier (Axon Instruments, Foster City, CA), and currents were evoked by applying voltage commands to a range of potentials in 10 mV steps from a holding potential of –20 mV. Drugs were added directly to the recording chamber. To elicit K_Ca_3.1 currents the K_Ca_3.1 opener 1-ethyl-2-benzimidazolinone (1-EBIO) (Tocris, Avonmouth, UK) was used at 100 µM. To block K_Ca_3.1 currents we used the specific K_Ca_3.1 channel blockers 1-[(2-chlorophenyl)diphenylmethyl]-1*H*-pyrazole (TRAM-34) (200 nM)[18] and ICA-17043 (Senicapoc)(100 nM)[19].

### Human myofibroblast proliferation

Human lung myofibroblasts (n=4) were seeded into 6 well plates, when 50% confluent cells were serum starved for 24h in serum-free medium. Cells were then stimulated with serum-free medium plus 0.1% DMSO, 10% FBS medium plus 0.1% DMSO, 10% FBS plus TRAM-34 20 nM and 10% FBS plus TRAM-34 200 nM. After 48h cells were mobilized with 0.1% trypsin/0.1% EDTA and counted used a standard haemocytometer. Cell viability was assessed by trypan blue exclusion. Results were counted by 2 blinded observers with excellent agreement (intra-class correlation of 0.969). All conditions were performed in duplicate.

### Wound healing assay

Human myofibroblasts were grown to confluence in 6 well plates. After 24 h in serum-free medium, 3 artificial wounds were scratched in each monolayer using a 200 µl pipette creating a linear cell-free area. After washing, TRAM-34 (20 and 200 nM) and ICA-17043 (Senicapoc, a gift from Icagen Inc, Durham, NC, USA) (10 and 100 nM) were added (final DMSO concentration 0.1%), and the cells stimulated with either 10 ng/ml bFGF or 10% FBS alone for 48 h. Control wells contained 0.1% DMSO and serum-free medium alone. Cells migrating and proliferating into the wound were observed and photographs taken over 48 h. Wound healing was analysed by measuring the area of the scraped wound using Cell F software by a blinded observer (Olympus, UK), and quantified as a percentage of the starting area of the wound scraped. Data presented represents the mean of the measurements from 2 different scratches. Results were measured by 2 blinded observers with excellent agreement (intra-class correlation of 0.969).

### Collagen Assay

Myofibroblasts were cultured in serum-free medium alone or 0.1% DMSO control, and stimulated with TGFβ1 10 ng/ml in the presence of DMSO control, TRAM-34 (20 and 200 nM) and ICA-17043 (10 and 100 nM) for 16 h. Soluble collagen released by myofibroblasts was quantified using the Sircol collagen assay (Biocolor, County Antrim, UK) according to the manufacturer’s instructions [20,21].

### Collagen Gel Contraction

Cells were pre-treated for 24 hours with serum-free media, 0.1% DMSO, TRAM-34 200 nM, ICA-17043 100 nM, TRAM-7 200 nM or TRAM-85 200 nM. Cells were detached and collagen gels were set up as described in [22]. TGFβ1 and bFGF were then added to appropriate wells to a final concentration of 10 ng/ml. Photographs were taken at 0 h and 22 h. The surface area was measured at each time point using ImageJ software (http://rsbweb.nih.gov/ij/) by 2 blinded observers with excellent agreement (intraclass correlation of 0.974).

### Calcium Imaging

Changes in [Ca^2+^]i were assessed with the use of a flurometric Ca^2+^ probe Fura-2 (Sigma-Aldrich). Myofibroblasts were grown on 35 mm fluorodish cell culture dishes (WPI, Hertfordshire, UK) and when 40% confluent loaded with Fura-2 for 45 min at 37°C in normal physiological saline solution (140 mmol/L NaCl, 5 mmol/L KCl, 2 mmol/L CaCl_2_, 1 mmol/L MgCl_2_, 10 mmol/L HEPES, pH 7.4 with NAOH) containing 5 µmol/L fura-2-acetoxymethyl ester and 2.5 mmol/L probenecid. The cells were then washed and [Ca^2+^]i recorded using the single cell recording system previously described [23]. Data acquisition occurred at a rate of 1 dual wavelength image every second and are presented as the 340 nm/380 nm ratio. 

### Statistical analysis

Experiments from an individual donor were performed either in duplicate or triplicate and a mean value was derived for each condition. Data distribution was tested for normality using the Kolmogorov-Smirnov test. For parametric data the 1-way ANOVA or repeated measures ANOVA for across-group comparisons was used followed by the appropriate multiple comparison post hoc test; otherwise an unpaired or paired t-test was used. Where appropriate chi-squared tests were used. For non-parametric data the Kruskal-Wallis test was used for across group comparisons with the Dunn’s multiple comparison post hoc test, or the Mann Whitney U test was used where there were two unpaired groups. GraphPad Prism for windows (version 6, GraphPad Software, San Diego California USA) was used for these analyses. A value of P<0.05 was taken to assume statistical significance.

## Results

### Characterization of human lung myofibroblasts

**Figure 1:**
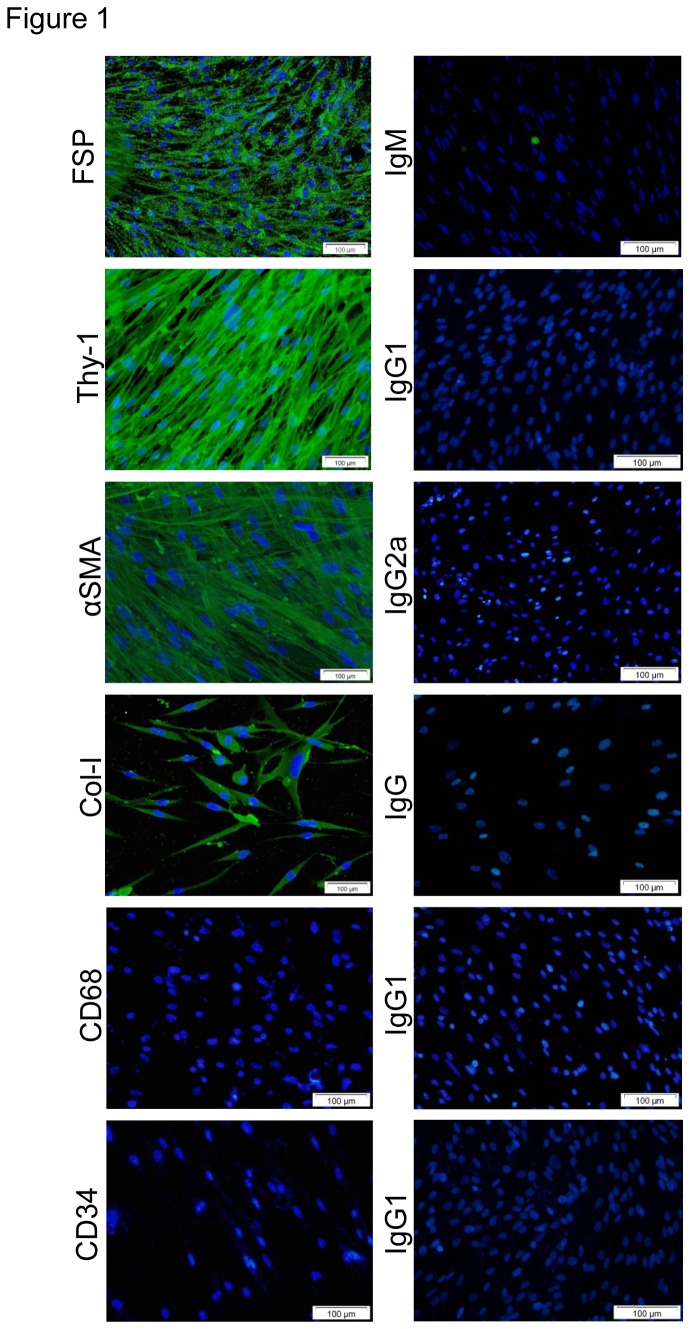
Characterization of human lung myofibroblasts by immunofluorescence. Primary human lung myofibroblast cultures between passages 4 and 5 were stained with myofibroblast markers. Representative images are shown for: anti-fibroblast surface protein (FSP) and the mouse isotype control IgM; anti-fibroblast antigen which recognises the fibroblast antigen (thy-1/CD90) and the mouse isotype control IgG1; α-smooth muscle actin, and the isotype control IgG2a; collagen type 1 antibody and rabbit isotype control IgG; CD68 cell staining was negative; and corresponding isotype control IgG3, indicating that there is no contamination of monocytes or macrophage cells; CD34 antibody shows negative staining as does the appropriate isotype control IgG1. Nuclei are stained with DAPI.

The clinical characteristics of the myofibroblast donors are listed in Table **1**. Cultured human lung myofibroblasts displayed the typical morphology of fibroblast-related cells with a spindle or stellate shape (Figure **1**). No differences in THY-1, 1B10 and αSMA expression were found between passages 2 and 4. At passage 4, >98% of cells from all donors expressed the fibroblast marker FSP, >97% expressed the fibroblast marker THY-1, and >99% expressed αSMA, confirming that these cells were almost exclusively myofibroblast in phenotype. Collagen 1 staining was present in 100% of cells (Figure **1**). There was no difference between NFC and IPF cells. No cells expressed the macrophage/monocyte marker CD68 or mesenchymal cell marker CD34. All isotype controls were negative (Figure **1**). 

**Table 1:** Clinical characteristics of NFC and IPF patients.

**Characteristic**	**IPF (N=9)**	**Non Fibrotic (N=8)**
**Sex (no. of subjects)**		
Male	8 (90%)	3 (37.5%)
Female	1 (10%)	5 (62.5)
**Age (yr)**		
Mean ( ± SEM)	59.77 ± 3.39	69.5 ± 3.3
Range	40 - 70	58 - 80
**Length of symptoms (yrs)**		
Mean ( ± SEM)	4.86 ± 1.38	NA
Range	1 - 11	NA
**Smoking (no. with >10 pack/years)**	7	5
**FEV_1_ (% predicted ± SEM)**	39.56 ± 3.675	112.9 ± 11.04
**FVC (% predicted ± SEM)**	45.63 ± 6.074	92.54 ± 10.51
**DLCO (% predicted ± SEM)**	26.63 ± 3	ND
**PA mean (% predicted ± SEM)**	28.38 ± 2.57	ND
**Treatments**		
Prednisone	7	0
Mycophenolate Mofetil	5	0
Azathioprine	2	0
Methylprednisolone sodium succinate	2	0
Tacrolimus	2	0

This table shows the clinical characteristics of the human lung myofibroblasts isolated from both the NFC (n=8) and IPF (n=9) donors. Key; NA – Not applicable, ND- Not done.

### Myofibroblasts express K_Ca_3.1 channel mRNA, which is up-regulated by TGFβ1

**Figure 2:**
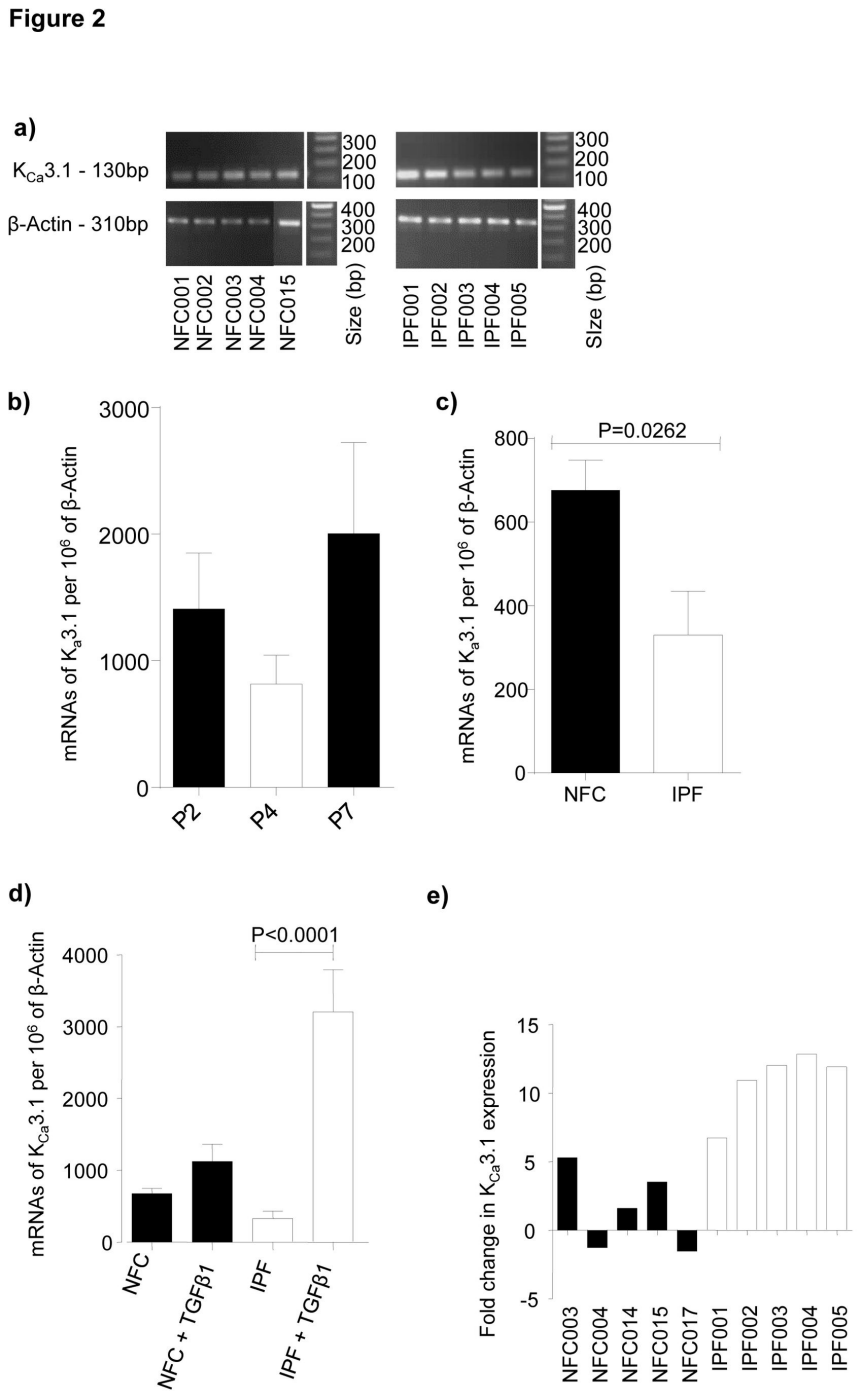
K_Ca_3.1 channel mRNA is expressed by myofibroblasts and upregulated following TGFβ1 stimulation. **a**) Products from quantitative real-time PCR for K_Ca_3.1 were visualized on a 1.5% agarose gel to confirm that only one product was amplified and that it was the correct size (130 bp). β-Actin was used as the normalizing control (310bp). **b**) In preliminary experiments we examined the mRNA expression levels of K_Ca_3.1 in the NFC donors at passages 2, 4 and 7 and again found no significant differences. To be sure that passage number had no effect all experiments were performed between passages 4 and 5. Data represent mean±SEM. **c**) Quantitative real-time PCR showed that K_Ca_3.1 mRNA expression was greater in NFC donors (n=5) than IPF donors (n=5), *P*=0.0262 (unpaired t test). **d**) K_Ca_3.1 mRNA expression increased after TGFβ1 stimulation (All groups; 1-way ANOVA, *P*<0.0001), NFC donors (n=5), IPF donors (n=5). There was a highly significant increase in IPF myofibroblasts following 24h of TGFβ1 stimulation. *P*<0.0001 (corrected by Bonferroni's multiple comparison test). **e**) Quantitative real-time PCR demonstrating the relative fold increase in NFC and IPF myofibroblasts after stimulation with TGFβ1. Following normalization with β-actin there was a relative fold increase in K_Ca_3.1 expression in all IPF donors. Results were calculated using the δδCT method. Data represented as ±SEM for Figure 1b,c and d.

Human lung myofibroblasts (n=5 NFC, n=5 IPF) expressed K_Ca_3.1 mRNA (Figure **2a**). No difference in K_Ca_3.1 mRNA expression was found between passages, but all experiments were performed between passages 4 and 5 for consistency (Figure **2b**). K_Ca_3.1 mRNA was significantly increased in cells from NFC compared to IPF donors (Figure **2c**). TGFβ1 (10 ng/ml) stimulation for 24h upregulated myofibroblast K_Ca_3.1 channel mRNA relative to β-actin, which was significantly greater in IPF compared NFC cells (Figure **2d**). K_Ca_3.1 mRNA was up-regulated at least 8 fold in every IPF donor after TGFβ1 exposure (Figure **2e**). 

### Myofibroblasts express K_Ca_3.1 protein

**Figure 3:**
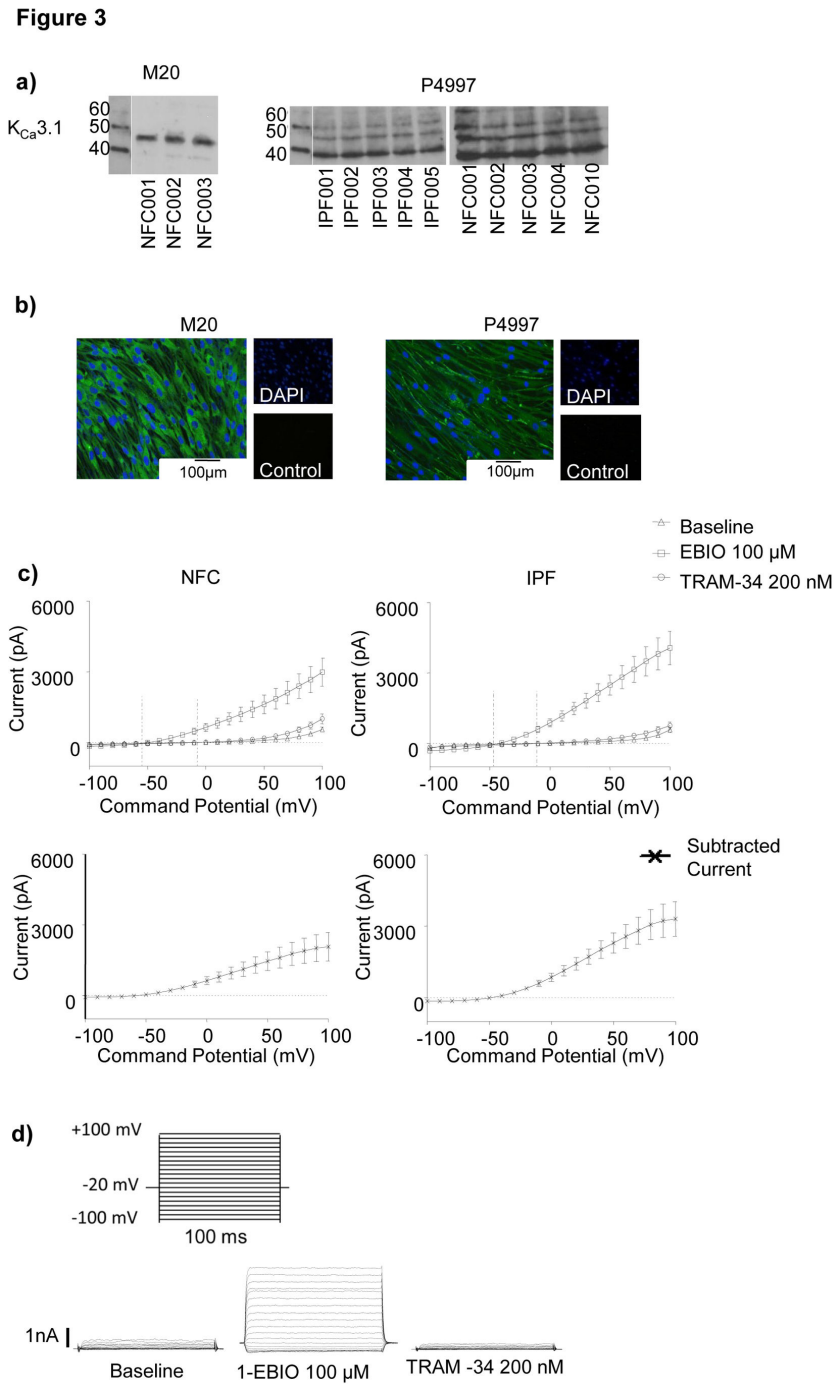
K_Ca_3.1 channel protein is present within myofibroblasts and K_Ca_3.1 channels are functional. **a**) Western blot of human lung myofibroblast lysates using 2 different K_Ca_3.1 channel antibodies, M20 and P4997. All images show a consistent band at the predicted size for the K_Ca_3.1 channel at 48 KDa in human lung myofibroblasts. An additional band at 53 kDa is present as described in other cell types. **b**) Example of immunofluorescent staining for K_Ca_3.1 in NFC myofibroblasts using M20 and P4997 antibodies. DAPI nuclear staining and negative rabbit isotype control IgG are shown. **c**) Whole-cell patch-clamp electrophysiology recordings of K_Ca_3.1 in NFC (n=14) and IPF (n=13) human lung myofibroblasts activated with 1-EBIO and blocked with TRAM-34 (200 nM). Upper panels: Mean ± SEM current voltage curves demonstrate a small outwardly rectifying current at baseline, and the IPF donors have a relatively small inwardly rectifying Kir current (confirmed by blocking with 10 µM barium, results not shown). Large currents with a negative reversal potential develop after the addition of the K_Ca_3.1 opener 1-EBIO (100 µM), which are blocked by the selective K_Ca_3.1 blocker TRAM-34 (200 nM). Lower panels: The subtracted (1-EBIO minus TRAM-34) TRAM-34-sensitive K_Ca_3.1 current. **d**) The voltage protocol and the raw current are demonstrated showing typical electrophysiological features of K_Ca_3.1 in a myofibroblast.

K_Ca_3.1 protein expression in human lung myofibroblasts was identified by Western blot (n=6 NFC, n=5 IPF). The predicted weight of K_Ca_3.1 is 48kDa, but larger forms of ~53kDa and several shorter splice variants exist [11,24-26]. Using two different anti-K_Ca_3.1 antibodies, M20 and P4997, a consistent band of ~48kDa was observed (Figure **3a**). M20 and P4997 also stained bands of 53kDa as described in human fibrocytes and airway smooth muscle cells [11,26], and 39kDa consistent with the presence of splice variants [25]. K_Ca_3.1 protein expression in myofibroblasts was also evident using immunofluorescent staining (Figure **3b**).

### Myofibroblasts express K_Ca_3.1 channel currents which are increased in IPF

At baseline, myofibroblasts from NFC and IPF donors demonstrated strong outwardly rectifying whole-cell currents, and frequent inwardly rectifying currents with features of the Kir2 family (confirmed by blocking with 10 µM barium, results not shown). Interestingly, 69% of IPF cells had Kir2-like currents at baseline as opposed to 42% of NFC cells (*P*=0.0007, chi squared).

K_Ca_3.1 currents in myofibroblasts were evoked using the K_Ca_3.1 opener 1-EBIO [27]. To block K_Ca_3.1 we used two distinct and selective K_Ca_3.1 channel blockers, TRAM-34 (*K*d for K_Ca_3.1 block 20 nM)[18] and ICA-17043 (Senicapoc)(*K*d ~10 nM)[19]. K_Ca_3.1 currents were not present at baseline but were frequently elicited following the addition of 1-EBIO (100 µM), and demonstrated the characteristic electrophysiological features of K_Ca_3.1 (Figure **3c and d**). In addition, the 1-EBIO-induced current was dose-dependently blocked by TRAM-34 (Figure **3c and d**) and ICA-17043 (not shown) with complete block at 200 nM and 100 nM respectively. DMSO vehicle (0.1% final concentration) had no effect (not shown). 

**Figure 4:**
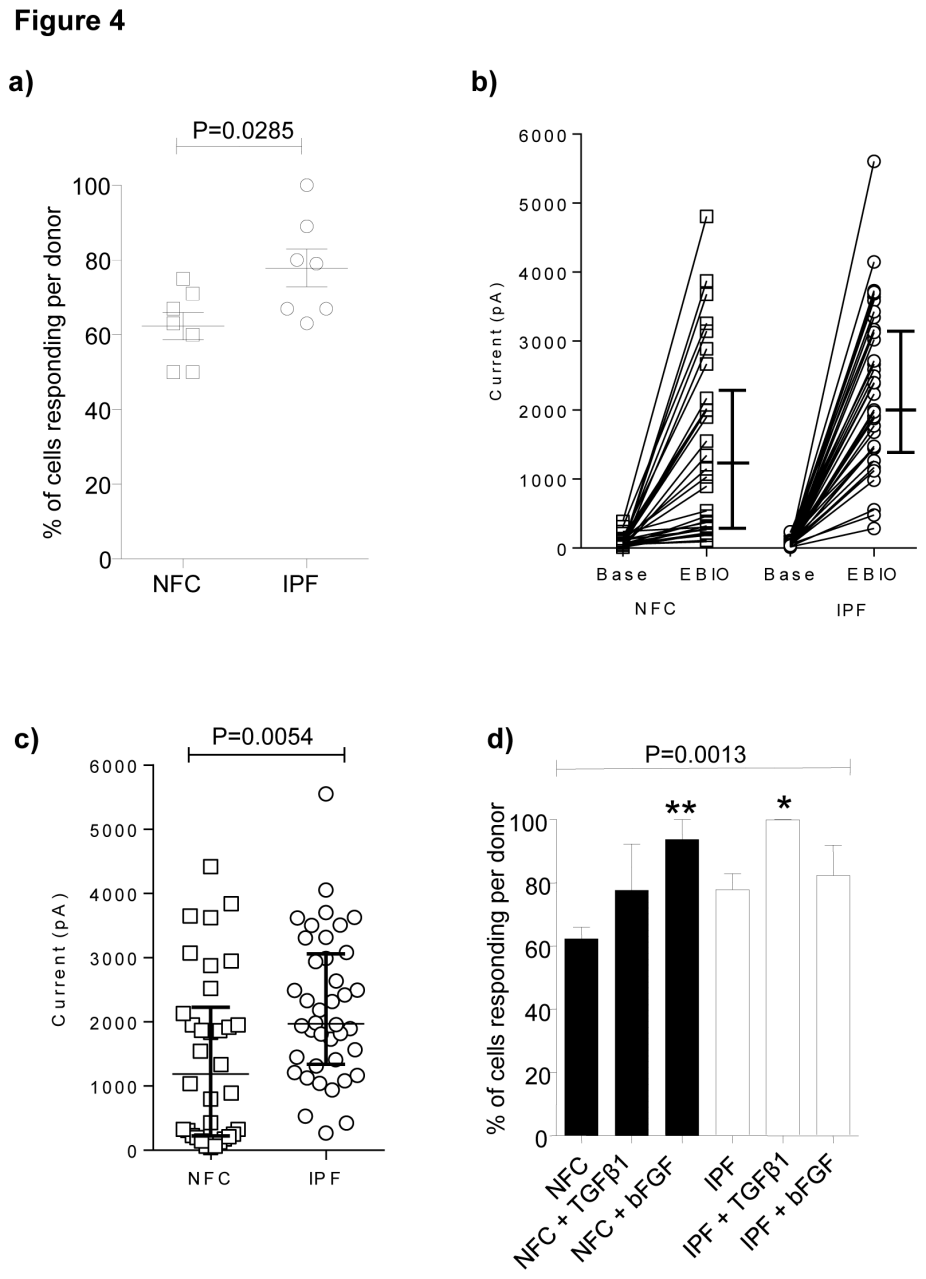
Functional K_Ca_3.1 channels demonstrate greater expression in IPF myofibroblasts compared to NFC myofibroblasts and channel expression is increased by pro-fibrotic growth factors. **a**) The mean percentage of IPF myofibroblasts per donor developing a K_Ca_3.1 current in response to 1-EBIO was significantly higher than in NFC myofibroblasts (*P*=0.0285, unpaired t-test). Data presented as mean±SEM. **b**) The whole-cell current at +40 mV before and after the addition of 1-EBIO in all responding NFC and IPF human lung myofibroblasts. Data presented as median and IQR. **c**) The subtracted (1-EBIO minus baseline) 1-EBIO-dependent K_Ca_3.1 current at +40 mV was significantly larger in IPF cells than in NFC cells (*P*=0.0054, Mann Whitney test). Data presented as median and IQR. **d**) The mean percentage of NFC and IPF myofibroblasts expressing K_Ca_3.1 currents increased after stimulation with TGFβ1 and bFGF (All groups; 1-way ANOVA, *P*=0.0013). The proportion of IPF cells responding to 1-EBIO after TGFβ1 stimulation was significantly higher (*P=0.0336, corrected by Bonferroni’s multiple comparisons test). Significantly more NFC cells responded to 1-EBIO following bFGF stimulation (**P=0.0035, corrected by Bonferroni’s multiple comparisons test). Data presented as mean±SEM.

The addition of 1-EBIO elicited K_Ca_3.1 currents in 59% of NFC myofibroblasts (n=7 donors) and 77% of IPF myofibroblasts (n=7 donors)(*P*=0.0411, Chi squared). Also, the proportion of myofibroblasts per donor responding to 1-EBIO was significantly increased in IPF compared to NFC cells (*P*=0.0285)(Figure **4a**), and the size of the currents induced by 1-EBIO was significantly greater in IPF compared to NFC cells (*P*=0.0054, Mann Whitney test) (Figure **4b and c**). Thus, functional K_Ca_3.1 channels were expressed more frequently and the currents were larger in myofibroblasts derived from IPF lung tissue compared to NFC tissue. 

Measurements of myofibroblast capacitance were unreliable due to the relatively large cell size, so it was not possible to calculate current density.

### The proportion of myofibroblasts expressing K_Ca_3.1 currents increases following mitogenic stimulation

After 24h of stimulation with 10 ng/ml basic fibroblast growth factor (bFGF), a greater proportion of myofibroblasts from NFC tissue expressed K_Ca_3.1 currents in comparison to un-stimulated cells (*P*=0.0035, corrected by Bonferroni’s multiple comparisons test), indicating increased functional channel expression. Similar results were seen in IPF myofibroblasts using TGFβ1 (*P*=0.0336, corrected by Bonferroni’s multiple comparisons test) (Figure **4d**).

### K_Ca_3.1 immunoreactivity is expressed in IPF parenchymal lung tissue

**Figure 5:**
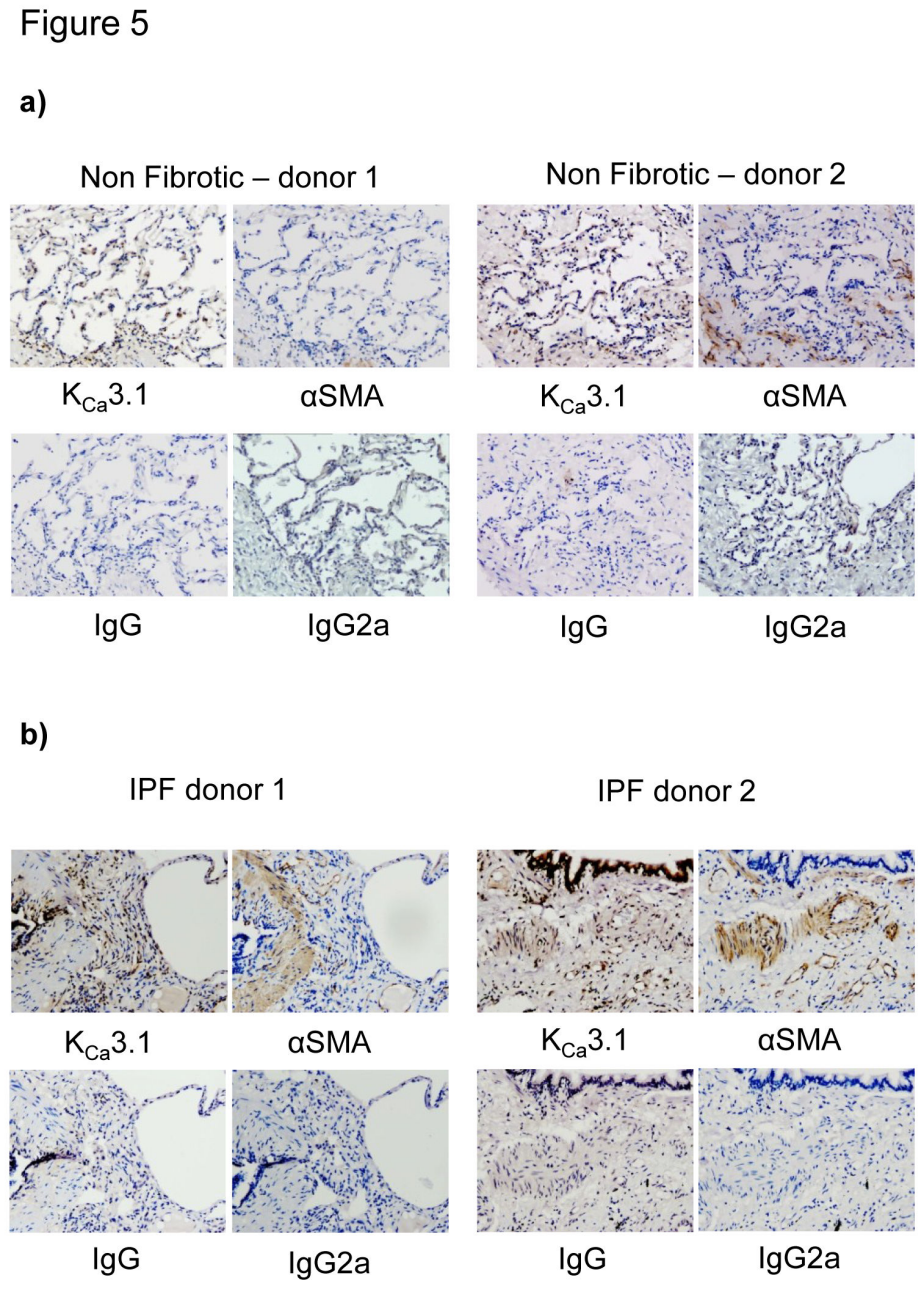
K_Ca_3.1 expression within human lung tissue of non-fibrotic and IPF patients. **a**) Representative K_Ca_3.1 and αSMA immunostaining of healthy lung parenchyma from two NFC tissue donors. All pictures are from sequential sections. Isotype controls are negative. **b**) Representative immunostaining of lung parenchyma from two IPF tissue donors demonstrating K_Ca_3.1 and αSMA immunostaining in areas of fibrosis. All pictures are from sequential sections. K_Ca_3.1 channel expression is particularly strong in the epithelium and within and surrounding areas positive for αSMA.

There was strong immunostaining for K_Ca_3.1 in NFC lung tissue (n=3), particularly in airway and alveolar epithelial cells, but also in cells within the interstitium including vessels and inflammatory cells (Figure **5a**). K_Ca_3.1 was also expressed strongly in areas of parenchymal fibrosis in IPF and co-localised with areas of αSMA positivity (n=5, IPF) (Figure **5b**). As there is no control for parenchymal fibrosis, we did not compare the magnitude of K_Ca_3.1 staining between NFC and IPF tissue. 

### K_Ca_3.1 channel inhibition inhibits myofibroblast proliferation

**Figure 6:**
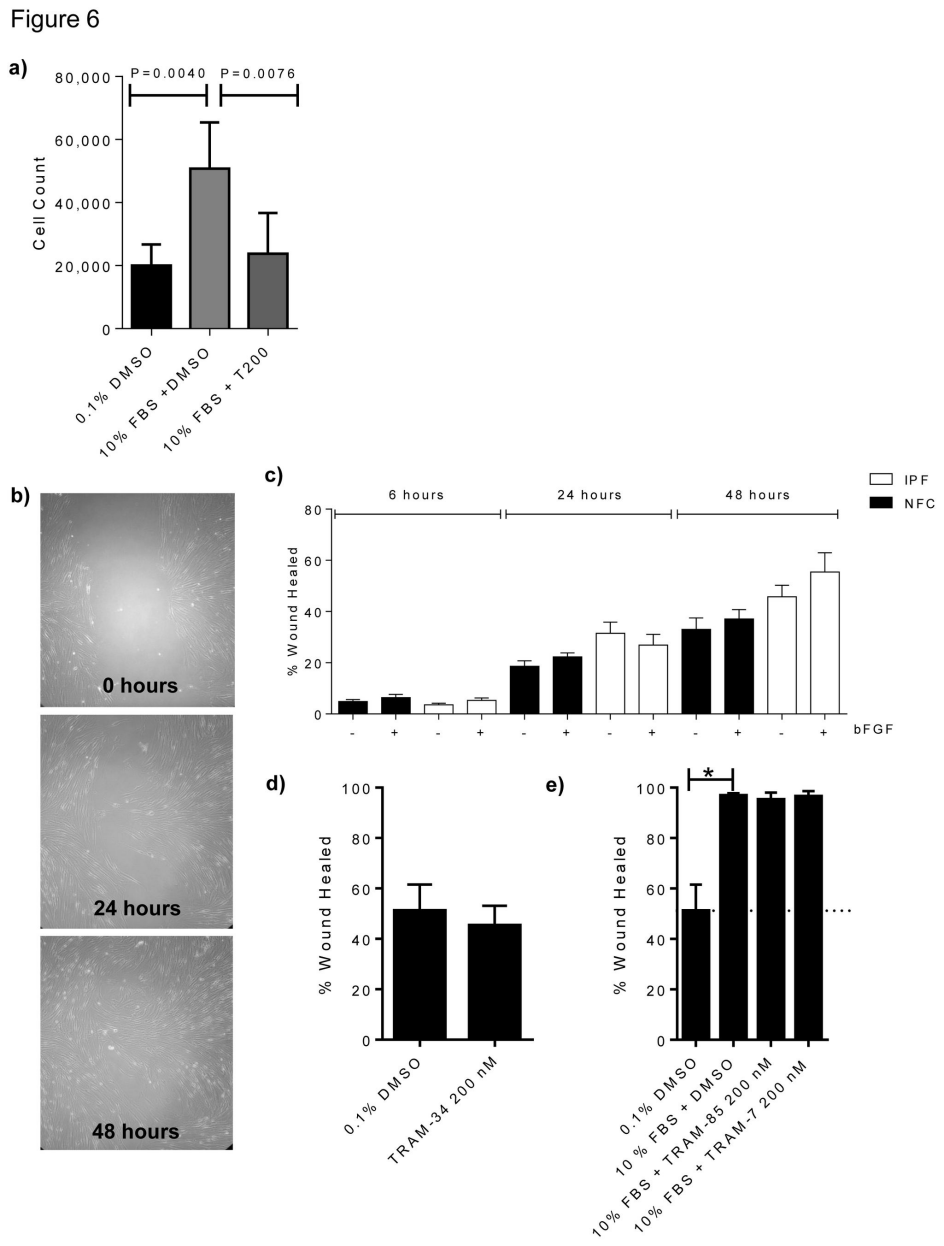
Myofibroblast proliferation is inhibited by K_Ca_3.1 channel block. Constitutive unstimulated wound healing is not altered by K_Ca_3.1 blockade, and growth factor-stimulated healing is not inhibited by TRAM-7 or TRAM-85 . **a**) Myofibroblast proliferation was increased following 48h of stimulation with FBS and significantly reduced by TRAM-34 (200 nM). **b**) An example of the wound created in a confluent monolayer of myofibroblasts in the wound healing assay and how it heals over the 48 hours. **c**) This graph displays that over the time course of the wound healing assay no significant differences were found between NFC and IPF donors in response bFGF, similar results were seen with FBS but results are not shown. **d**) TRAM-34 does not inhibit wound healing in the absence of mitogenic stimulation. **e**) FBS significantly increases wound healing (*P=0.0168, Paired t-test) but the molecules TRAM-7 and TRAM-85 which are structurally related to TRAM-34 do not have K_Ca_3.1 channel-blocking activity and do not inhibit mitogen-dependent wound healing. Data represent mean±SEM for all figures a, c, d and e.

Stimulation with 10% FBS for 48h significantly increased myofibroblast proliferation over control (*P*=0.0040, corrected by Bonferroni’s multiple comparisons test), in both NFC and IPF donors, and to the same extent. However, K_Ca_3.1 blockade with TRAM-34 (200 nM) inhibited FBS-induced proliferation (*P*=0.0076, corrected by Bonferroni’s multiple comparisons test)(Figure **6a**).

### Selective pharmacological blockade of K_Ca_3.1 attenuates wound healing

Myofibroblast wound healing was assessed in a 2-D scratch assay (Figure **6b**). Myofibroblasts stimulated with 10% FBS showed enhanced wound repair compared to media alone (*P*<0.0001, paired t-test). There was no difference between NFC- and IPF-derived cells and all data are pooled (Figure **6c**). Wound healing in the absence of FBS or bFGF was not affected by K_Ca_3.1 blockade (Figure **6d**) and no inhibition of wound healing was seen with two structurally related molecules without channel blocking activity, TRAM-85 and TRAM-7 (Figure **6e**)[13,28].

**Figure 7:**
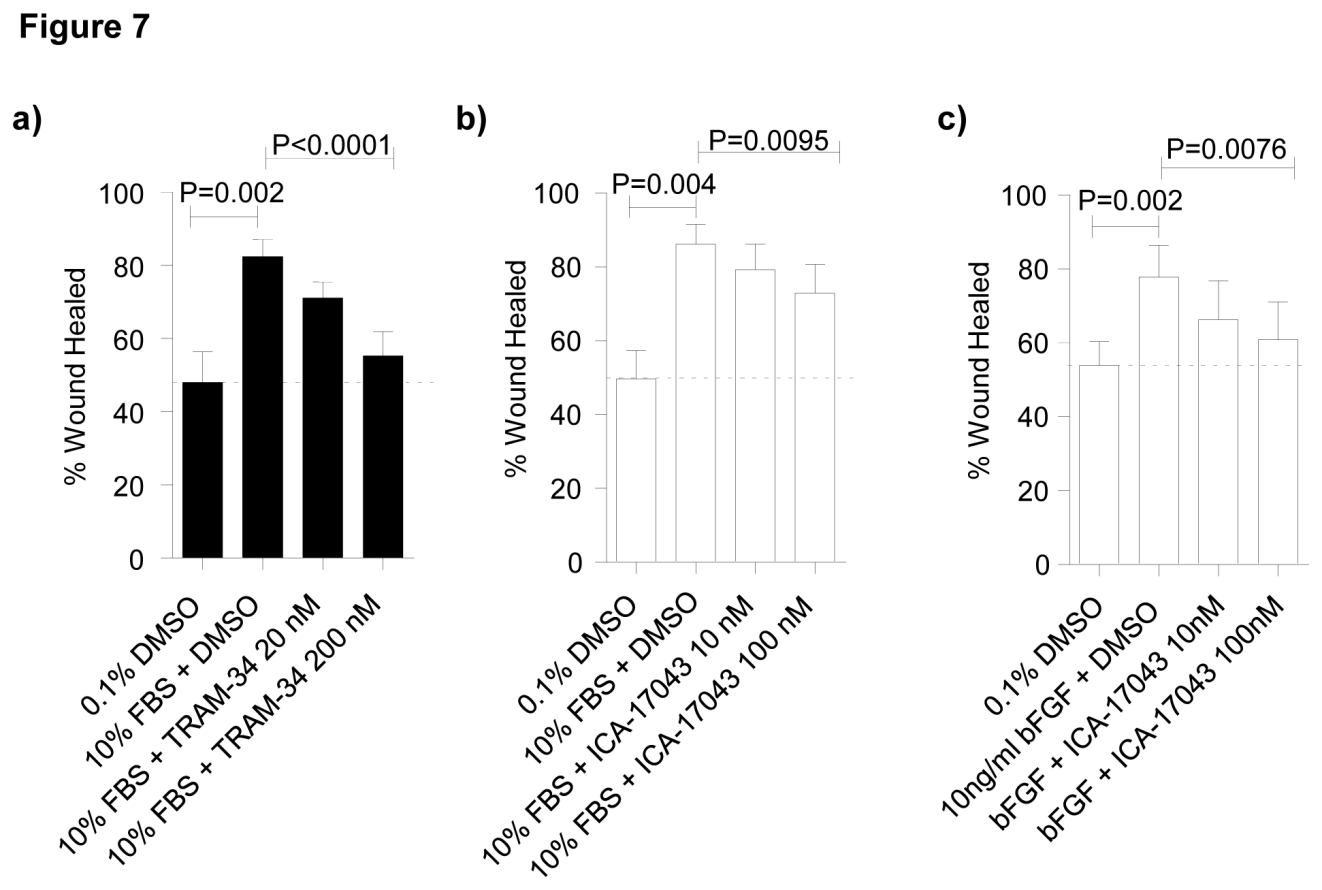
Blocking K_Ca_3.1 with TRAM-34 and ICA-17043 induces a dose-dependent attenuation of myofibroblast wound healing. **a**) and **b**) Myofibroblasts stimulated with 10% FBS and 0.1% DMSO vehicle control, showed accelerated wound healing in comparison to 0.1% DMSO alone (*P*=0.002, and P=0.004 respectively, paired t-test). There was a dose-dependent decrease in FBS-induced wound healing over 48h in the presence of either **a**) TRAM-34 (20 nM and 200 nM) (*P*<0.0001, repeated measures ANOVA) or **b**) ICA-17043 (10 nM and 100 nM) (*P*=0.0095, repeated measures ANOVA). **c**) Myofibroblast wound healing in response to 10 ng/ml bFGF and 0.1% DMSO stimulation at 48 h was increased in comparison to media alone (*P=0.002, paired t-test). There was a dose-dependent decrease in wound healing over the 48 h in the presence ICA-17043 (*P*=0.0076, Repeated measures ANOVA). Data represented as mean±SEM for all the above figures.

FBS-induced wound repair was dose-dependently attenuated by both TRAM-34 and ICA-17043 (repeated measures ANOVA, *P*<0.0001 and *P*=0.0095 respectively)(Figure **7a and b**). Thus, in 10% FBS, total wound healing decreased by (mean±SEM) 30.6±5.4% with TRAM-34 200 nM and by 17.2±5.9% with ICA-17043 100 nM; this was equivalent to inhibition of the FBS-dependent response by 75.7±10.9% and 47.9±15.3% respectively. 

Compared to unstimulated cells, bFGF (10ng/ml) also increased myofibroblast wound healing over 48h in both NFC and IPF donors (*P*=0.002, paired t-test). bFGF-induced wound repair was dose-dependently attenuated by ICA-17043 (repeated measures ANOVA, *P*=0.0076)(Figure **7c**). At 48 h, total wound healing was reduced by 24.4±6.6% in the presence of ICA-17043 100 nM compared to DMSO control (equivalent to inhibition of the FGF-dependent response by 63.4±13.4%). In summary, mitogen-induced wound healing of human lung myofibroblasts is attenuated by K_Ca_3.1 channel block. 

### TGFβ1-dependent collagen production in human myofibroblasts is attenuated by K_Ca_3.1 blockade

**Figure 8:**
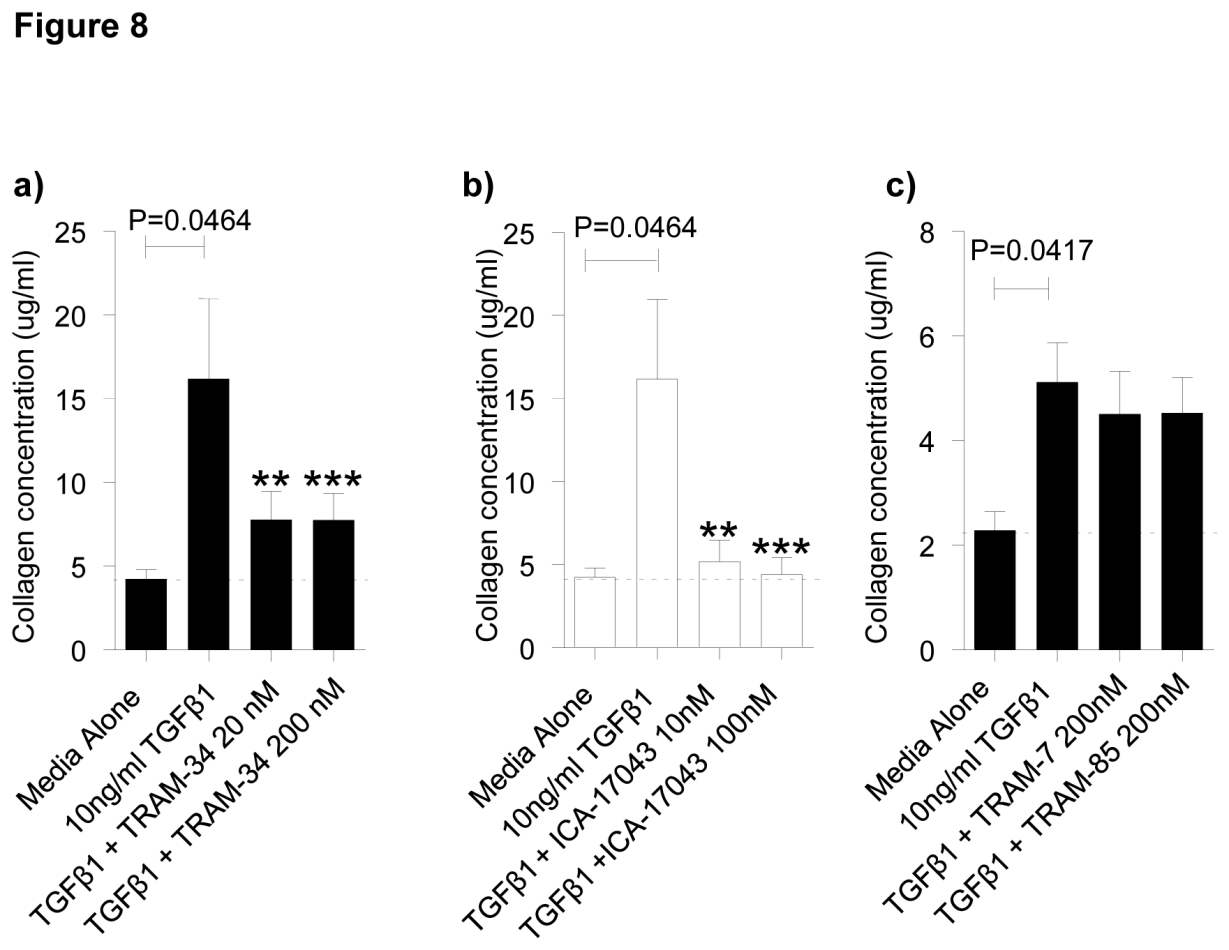
Blocking K_Ca_3.1 channels decreases TGFβ1-dependent myofibroblast collagen secretion. **a**) and **b**) Collagen secretion was increased in myofibroblasts following TGFβ1-dependent simulation in both IPF donors (n=4) and NFC donors (n=4), *P*=0.0464, paired t-test (data shown is pooled IPF and NFC which did not differ). **a**) This TGFβ1-induced increase was inhibited by TRAM-34 20 nM and 200 nM (*P*=0.0161, repeated measures ANOVA) and there were significant differences found between TGFβ1 and TRAM-34 20 nM (**P=0.0473, corrected by Bonferroni’s multiple comparisons test) and between TGFβ1 and TRAM-34 200 nM (***P=0.0469, corrected by Bonferroni’s multiple comparisons test). **b**) TGFβ1-dependent collagen secretion was also inhibited by ICA-17043 (*P*=0.0038, repeated measures ANOVA), and there were significant differences found between TGFβ1 and ICA-17043 10 nM (**P=0.0067, corrected by Bonferroni’s multiple comparisons test) and between TGFβ1and ICA-17043 100 nM (***P=0.0039, corrected by Bonferroni’s multiple comparisons test). **c**) TGFβ1 increased collagen secretion in myofibroblasts (*P=0.0417, paired t-test.), however no inhibition of collagen secretion was evident with TRAM-7 or TRAM-85 (n=3). Data represented as mean±SEM for all the above figures.

Myofibroblasts from both IPF and NFC lung secreted similar amounts of collagen in response to TGFβ1 (10 ng/ml). This was markedly attenuated by both TRAM-34 and ICA-17043 (Figure **8a and b**), but was not affected by TRAM-85 or TRAM-7 (Figure **8c**).

### K_Ca_3.1 inhibition attenuates TGF-β1 and bFGF induced myofibroblast contraction

**Figure 9:**
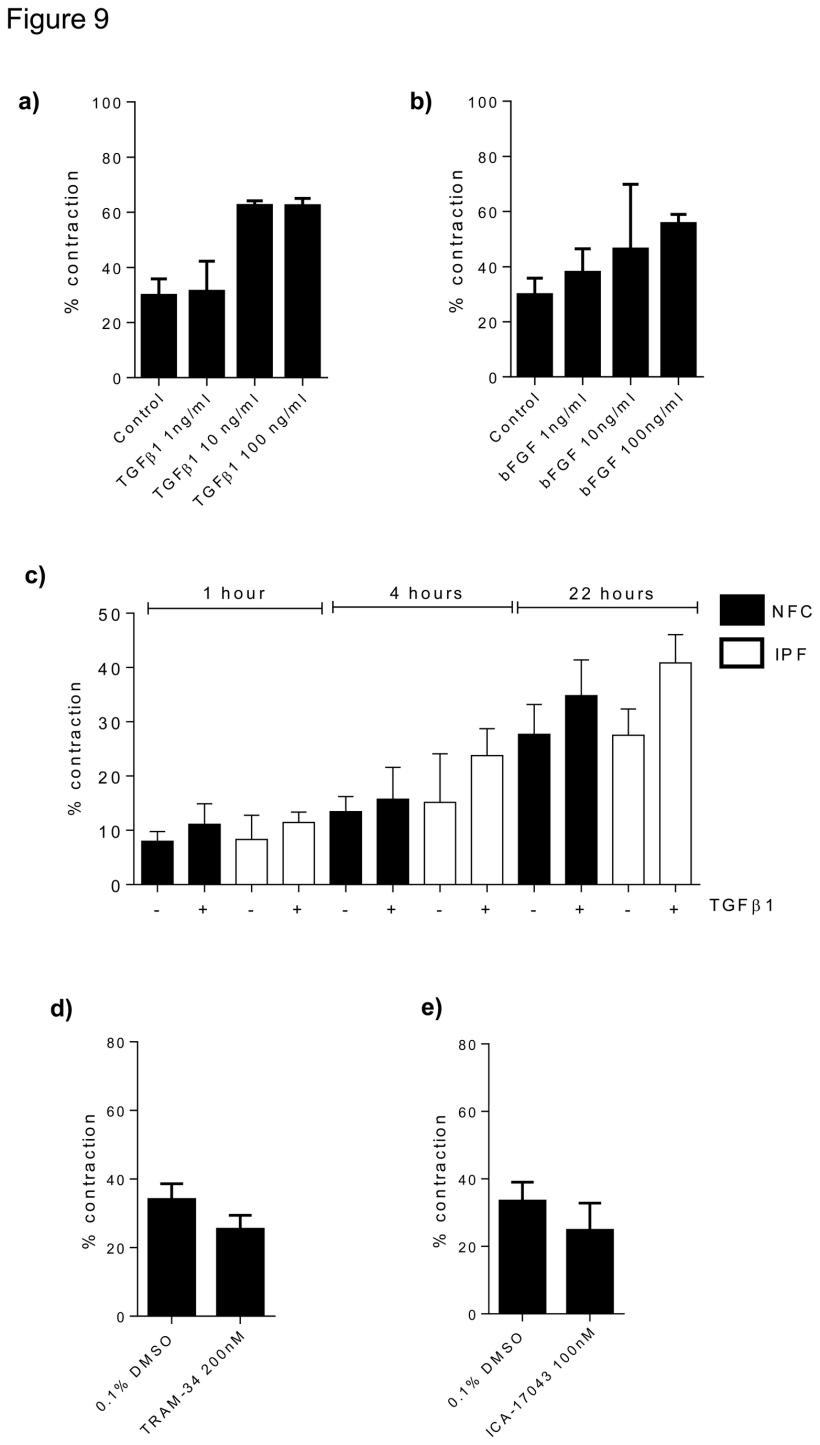
Constitutive unstimulated myofibroblast contraction is not altered by K_Ca_3.1 blockade, and 10 ng/ml of TGFβ1 and bFGF is optimal concentration. (a and b) dose response was performed before beginning experiments which confirmed 10 ng/ml as optimal concentration to use for both TGFβ1 and bFGF in the myofibroblast contraction assay, n=2.c) demonstrates that at the different time points of the contraction assay there were no significant difference between the response of the NFC and IPF cells to TGFβ1. At 1 hour NFC n=4 and IPF n=3, 4 hours NFC n=3, IPF n=2 and 22 hours NFC n=4 and IPF n=5. d) TRAM-34 does not significantly inhibit myofibroblast contraction in the absence of mitogenic stimulation, n=6. e) ICA-17043 does not significantly inhibit myofibroblast contraction in the absence of mitogenic stimulation, n=5. Data represent mean±SEM for all the above figures.

**Figure 10:**
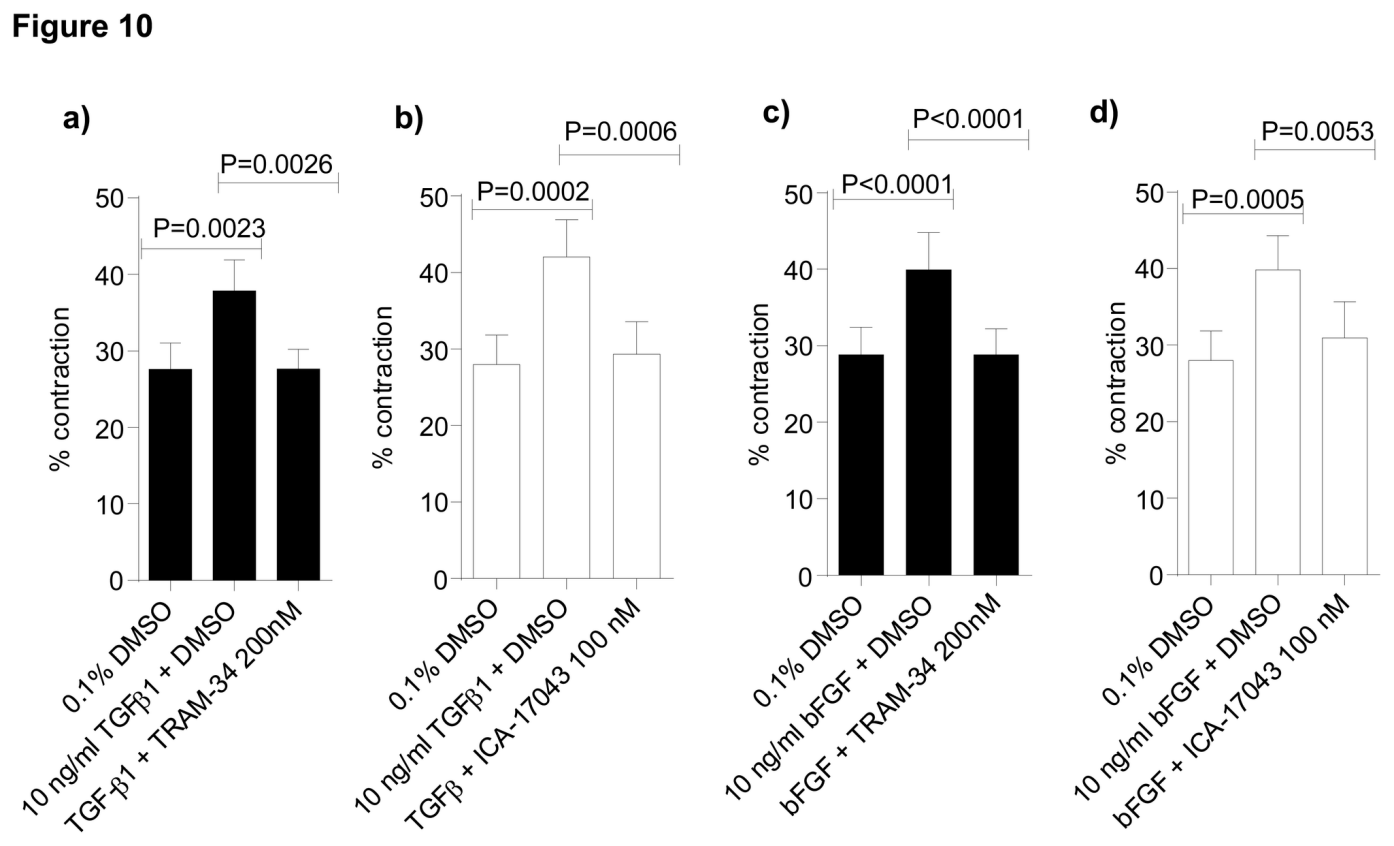
K_Ca_3.1 inhibition TGFβ1 and bFGF-induced myofibroblast contraction. **a**) Myofibroblast collagen gel contraction was increased following TGFβ1 stimulation and this was inhibited by TRAM-34 200 nM in both IPF (n=3) and NFC (n=3) donors (data shown is pooled IPF and NFC which did not differ, n=6)(*P*=0.0014, repeated measures ANOVA, *P*=0.0023 for TGFβ1 compared to control, *P*=0.0026 for TRAM-34 compared to TGFβ1 corrected by Bonferroni’s multiple comparisons test). **b**) TGFβ1-dependent myofibroblast collagen gel contraction was also inhibited by ICA-17043 100 nM (n=6) (All groups; repeated measures ANOVA, *P*=0.0002, TGFβ1 versus control, *P*=0.0002 and for ICA-17043 versus TGFβ1, *P*=0.0006, corrected by Bonferroni’s multiple comparisons test). **c**) Similarly, myofibroblast collagen gel contraction was increased following bFGF stimulation and was also inhibited with 24h pre-treatment with TRAM-34 200 nM (All groups; repeated measures ANOVA *P*<0.0001, for bFGF compared to control, *P*<0.0001, and for TRAM-34 compared to bFGF, *P*<0.0001 (corrected by Bonferroni’s multiple comparisons test). **d**) Similarly, 24h pre-treatment with ICA-17043 100 nM significantly reduced bFGF-dependent myofibroblast collagen gel contraction (*P*=0.0007, repeated measures ANOVA) (*P*=0.0005 for bFGF versus control and *P*=0.0053 for ICA-17043 versus bFGF, corrected by Bonferroni’s multiple comparisons test). Data represented as mean±SEM for all the above figures.

Myofibroblasts cultured in collagen gels from both IPF and NFC donors contracted following TGFβ1 stimulation and bFGF stimulation (Figure **9**). After dose response experiments were performed, 10 ng/ml chosen as the optimal concentration for each growth factor (Figure **9a and b**). There was no difference between NFC- and IPF-derived cells (Figure **9c**,) and all data are therefore pooled. TGFβ1 stimulation increased myofibroblast contraction from (mean±SEM) 30.6±3.6% to 48.6±2.4%, and bFGF stimulation increased myofibroblast contraction from 31.7±3.8% to 51.2±4.8%. Compared to DMSO control, pre-treatment for 24 h in the presence of either TRAM-34 200 nM or ICA-17043 100 nM almost completely inhibited both TGFβ1- and bFGF-stimulated myofibroblast contraction (Figure **10a-d**). In contrast, the control molecules TRAM-7 and TRAM-85 were without effect (not shown).

TRAM-34 and ICA-17043 did not inhibit baseline constitutive myofibroblast contraction significantly in the absence of growth factors (Figure **9d and e**), indicating that TRAM-34 and ICA-17043 only inhibit TGFβ1- or bFGF-dependent myofibroblast contraction. 

### K_Ca_3.1 regulates myofibroblast pro-fibrotic functions by inhibiting TGFβ1 induced increases in [Ca^2+^]i

**Figure 11:**
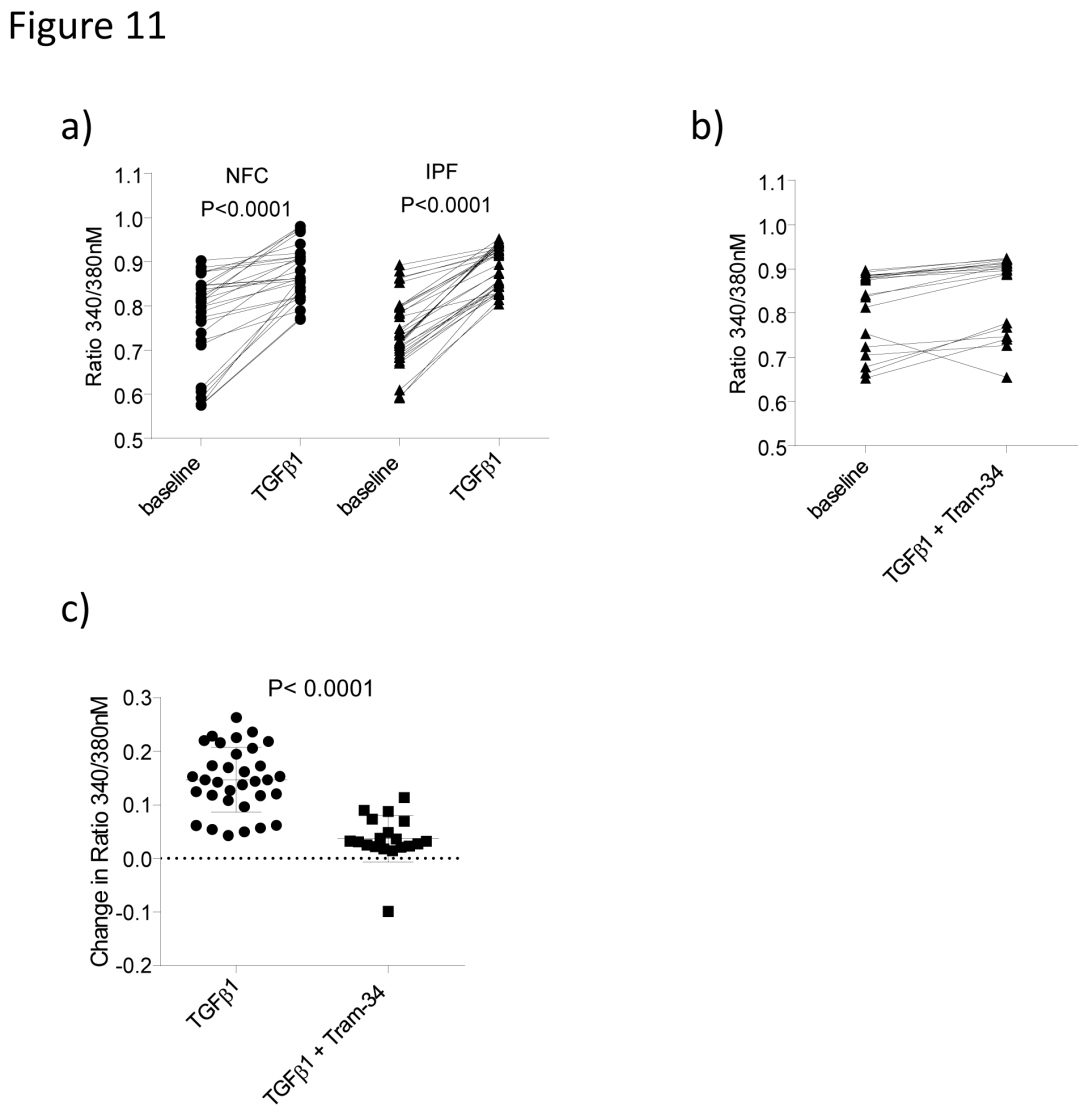
TGFβ1 induces a rise in intracellular calcium which is inhibited by pharmacological blockade of K_Ca_3.1. Upon stimulation with TGFβ1 (10 ng/ml) both NFC (n=4 donor, n=28 cells) and IPF (n=3 donors, n=33 cells) donors showed a significant rise in [Ca^2+^]i concentration as displayed the ratio 340/380 nm, *P*<0.0001 for both, paired t-test. **b**) In IPF only (n=3 donors and n=19 cells), TRAM-34 (200 nM) was added 5 minutes prior to treatment with TGFβ1, which inhibited the rise in [Ca^2+^]i typically seen upon TGFβ1 stimulation. **c**) The difference in [Ca^2+^]i following the different treatments were calculated and TRAM-34 significantly decreased the change in [Ca^2+^]i, *P*<0.0001, Mann Whitney t-test.

Open K_ca_3.1 channels hyperpolarise plasma membranes which in turn promotes Ca^2+^ influx [12]. This is believed to account for the ability of K_Ca_3.1 inhibition to attenuate many diverse cell responses. To investigate the underlying mechanism behind the attenuation of pro-fibrotic myofibroblast function with K_Ca_3.1 blockers, we therefore investigated whether changes in [Ca^2+^]i occur following stimulation with TGFβ1. TGFβ1 (10 ng/ml) elicited an immediate, rapid rise in [Ca^2+^]i in 100% of IPF cells and 53% of NFC cells. There was a significant increase in [Ca^2+^]i for both IPF (n=33, P<0.0001) and NFC (n=28, P<0.0001)(Figure **11a**). In the IPF donors only, TRAM-34 was added 5 minutes prior to the addition of TGFβ1 which inhibited the TGFβ1 induced rise in [Ca^2+^]i (Figure **11b**). The change in [Ca^2+^]i following TGFβ1 stimulation was significantly reduced with 5 minutes pre-treatment of TRAM-34 200 nM compared to controls, P<0.0001 (Figure **11c**). Cells were also treated with 0.1% DMSO control which had no effect on subsequent TGFβ1-dependent increase in [Ca^2+^]_I_, data no shown.

## Discussion

There are three major, novel findings from this study. Firstly we have performed the first detailed electrophysiological analysis of primary human parenchymal myofibroblasts from both NFC and IPF lung, and show for the first time that these cells express the Ca^2+^-activated K^+^ channel K_Ca_3.1 in both health and disease. Secondly we demonstrate that pharmacological blockade of K_Ca_3.1 inhibits human lung myofibroblast pro-fibrotic activity such as proliferation wound healing, collagen secretion and growth factor-dependent contraction. Thirdly, TGFβ1-induced myofibroblast pro-fibrotic function is associated with K_Ca_3.1-dependent regulation of [Ca^2+^]i. These findings highlight the importance of the K_Ca_3.1 channel and Ca^2+^ signaling in the growth factor-dependent pro-fibrotic functions of human lung myofibroblasts. 

The widely accepted markers of a myofibroblast are that it co-expresses fibroblast-associated markers and αSMA fibres [6]. The cells studied in these experiments were predominantly of a myofibroblast phenotype, a finding consistent with the parenchymal lung myofibroblast phenotype described by others [29,30]. 

K_Ca_3.1 channel mRNA, protein and functional channels were expressed in myofibroblasts derived from both NFC and IPF donors. Interestingly, K_Ca_3.1 currents were present more frequently in IPF lung myofibroblasts and these currents were larger when compared to NFC lung myofibroblasts. This might reflect K_Ca_3.1 up-regulation driven by the initiating disease insult in vivo, but the cells studied had been through 4 passages of culture, raising the possibility of an underlying disease-predisposing difference in myofibroblast K_Ca_3.1 activity in patients with IPF. Surprisingly, although K_Ca_3.1 currents were increased in IPF myofibroblasts, K_Ca_3.1 mRNA was decreased. However, chronic exposure of cells to the K_Ca_3.1 opener 1-EBIO down-regulates K_Ca_3.1 mRNA expression [31,32], suggesting a negative-feedback mechanism which is perhaps operative here. Whether the increased K_Ca_3.1 plasma membrane expression in IPF myofibroblasts represents increased trafficking to the cell membrane or decreased turnover requires further study.

TGFβ1 is a key mediator of fibrotic diseases, playing an important role in myofibroblast differentiation [33], matrix protein production [34], and possibly epithelial-mesenchymal transition [35]. TGFβ1 stimulation increased myofibroblast K_Ca_3.1 mRNA expression, which was greatest in IPF-derived myofibroblasts, and increased the frequency of cells expressing functional membrane channels. bFGF, another key mediator in fibrotic diseases, is a potent chemoattractant and mitogen for myofibroblasts, and regulates extracellular matrix production[34]. bFGF stimulation also increased the frequency of cells expressing K_Ca_3.1 currents (mRNA expression was not studied). These observations are in keeping with bFGF- and TGFβ1-dependent K_Ca_3.1 upregulation in several cell types [36].

The upregulation of functional K_Ca_3.1 channels by both TGFβ1 and bFGF suggests that the biological effects of these growth factors might rely heavily on K_Ca_3.1 channel activity. This is supported by the observations that serum-dependent proliferation, serum- and bFGF-dependent myofibroblast wound healing, TGFβ1-dependent collagen secretion, and both bFGF and TGFβ1-dependent myofibroblast contraction were attenuated by two distinct and specific K_Ca_3.1 blockers, TRAM-34 and ICA-17043. Importantly, these ion channel blockers inhibited these cell processes at physiologically relevant concentrations. Thus it takes 5-10x the *K*_d_ to inhibit almost all channels (*K*_d_=concentration producing 50% block). The *K*_d_ for TRAM-34 is 20 nM [11] and for ICA-17043 6-10 nM [10]. At 10x the *K*_d_ for both blockers, myofibroblast wound healing, collagen secretion and contraction were significantly attenuated. At these concentrations both drugs are specific and not known to affect other ion channels, receptors or transporters [13]. Furthermore, the effects of TRAM-34 and ICA-17043 on myofibroblast biology were not mimicked by TRAM-7 or TRAM-85, two molecules of similar structure to TRAM-34 which do not block K_Ca_3.1 ion channels [13,28].

Upon TGFβ1 stimulation human lung myofibroblast exhibited a rise in [Ca^2+^]i concentrations which was blocked by TRAM-34. This demonstrates how both membrane hyperpolarization and Ca^2+^ influx is critical for myofibroblast function in keeping with observations in other cells types [10,11,26], and provides mechanistic insight for the role of K_Ca_3.1 in lung myofibroblast responses. 

Although K_Ca_3.1 channels were more highly expressed in IPF myofibroblasts than in NFC cells, the functional responses of the cells to growth factors were largely similar irrespective of their source. This is perhaps not surprising because membrane hyperpolarization can be achieved with as few as 12 channels per cell [32], meaning that Ca^2+^ dependent cell processes can proceed with very low channel expression. Similar shifts in membrane potential in NFC and IPF myofibroblasts are evident in Figure **3c**. However, the presence of significantly more channels in IPF myofibroblasts suggests that it will be more difficult for a physiological process to overcome the effects of the increased channel activity. This however does not apply to channel blockers which will inhibit 10 or 1000 channels equally effectively. Therefore pharmacological K_Ca_3.1 blockers are attractive targets as they block all channels and will potentially attenuate pro-fibrotic responses as shown in this study.

K_Ca_3.1 was expressed in both NFC and IPF lung tissue, supporting the relevance of our in vitro findings. In IPF tissue, K_Ca_3.1 was not only expressed in areas of αSMA positivity, but also highly expressed in alveolar epithelial cells, vessels and inflammatory cells. In addition to alveolar epithelial cells, both mast cells and fibrocytes are implicated in IPF progression and express K_Ca_3.1 [10,26]. Targeting K_Ca_3.1 in IPF may therefore have additional benefits over and above those achieved through the inhibition of myofibroblast function.

Our data indicate that the K_Ca_3.1 channel may play a key role in the development of lung fibrosis, in both IPF and other lung disorders. K_Ca_3.1 is an attractive pharmacological target as it appears to play a minor role in healthy physiology, but contributes significantly to tissue remodeling and fibrosis [37]. It has been suggested that K_Ca_3.1 opening contributes to pulmonary vascular relaxation in rats[38], but this was not the case in human pulmonary vessels[39]. Importantly, K_Ca_3.1 knockout mice are viable, of normal appearance, produce normal litter sizes, and exhibit rather mild phenotypes [13]. High doses of TRAM-34 administered to rodents over many weeks are well tolerated [13] and the orally available K_Ca_3.1 blocker, ICA-17043, has been administered to humans in phase 2 and 3 trials of sickle cell disease with minor side effects [40]. There is therefore the potential for the rapid investigation of K_Ca_3.1 blockade in clinical trials of IPF and other fibrotic lung diseases.
