# Correction: The K^+^ Channel K_Ca_3.1 as a Novel Target for Idiopathic Pulmonary Fibrosis 

**DOI:** 10.1371/annotation/790e86f8-3506-49d6-b7d0-7dbbc580d808

**Published:** 2014-01-16

**Authors:** Katy M Roach, Stephen Mark Duffy, William Coward, Carol Feghali-Bostwick, Heike Wulff, Peter Bradding

In the author byline, the name of the first author is given incorrectly. The correct name is: Katy M Roach.

